# MAN1B1 Deficiency: An Unexpected CDG-II

**DOI:** 10.1371/journal.pgen.1003989

**Published:** 2013-12-12

**Authors:** Daisy Rymen, Romain Peanne, María B. Millón, Valérie Race, Luisa Sturiale, Domenico Garozzo, Philippa Mills, Peter Clayton, Carla G. Asteggiano, Dulce Quelhas, Ali Cansu, Esmeralda Martins, Marie-Cécile Nassogne, Miguel Gonçalves-Rocha, Haluk Topaloglu, Jaak Jaeken, François Foulquier, Gert Matthijs

**Affiliations:** 1 Center for Human Genetics, University of Leuven, Leuven, Belgium; 2 Center for Metabolic Diseases, University Hospital Gasthuisberg, Leuven, Belgium; 3 Centro de Estudio Metabalopatías Congénitas, Faculdad de Ciencias Médicas, Universidad Nacional de Córdoba, Hospital de Niños de la Santísima Trinidad, Córdoba, Argentina; 4 Institute of Chemistry and Technology of Polymers, CNR, Catania, Italy; 5 Clinical & Molecular Genetics Unit, Institute of Child Health, University College and Great Ormond Street Hospital for Children NHS Trust, London, United Kingdom; 6 Unidade de Genética Médica, Departamento de Genética Humana, Centro de Genética Médica - Dr. Jacinto Magalhães - INSA, IP. Porto, Portugal; 7 Gazi University Faculty of Medicine, Department of Paediatric Neurology, Besevler/Ankara, Turkey; 8 Unidade de Doenças Metabólicas, Hospital de Crianças Maria Pia, Porto, Portugal; 9 Université Catholique de Louvain, Cliniques Universitaires Saint-Luc, Brussels, Belgium; 10 Department of Child Neurology, Hacettepe University Children's Hospital, Ankara, Turkey; 11 Structural and Functional Glycobiology Unit, UMR CNRS/USTL 8576, IFR 147, University of Lille 1, Villeneuve d'Ascq, France; Max Planck Institute for Molecular Genetics, Germany

## Abstract

Congenital disorders of glycosylation (CDG) are a group of rare metabolic diseases, due to impaired protein and lipid glycosylation. In the present study, exome sequencing was used to identify *MAN1B1* as the culprit gene in an unsolved CDG-II patient. Subsequently, 6 additional cases with MAN1B1-CDG were found. All individuals presented slight facial dysmorphism, psychomotor retardation and truncal obesity. Generally, MAN1B1 is believed to be an ER resident alpha-1,2-mannosidase acting as a key factor in glycoprotein quality control by targeting misfolded proteins for ER-associated degradation (ERAD). However, recent studies indicated a Golgi localization of the endogenous MAN1B1, suggesting a more complex role for MAN1B1 in quality control. We were able to confirm that MAN1B1 is indeed localized to the Golgi complex instead of the ER. Furthermore, we observed an altered Golgi morphology in all patients' cells, with marked dilatation and fragmentation. We hypothesize that part of the phenotype is associated to this Golgi disruption. In conclusion, we linked mutations in *MAN1B1* to a Golgi glycosylation disorder. Additionally, our results support the recent findings on MAN1B1 localization. However, more work is needed to pinpoint the exact function of MAN1B1 in glycoprotein quality control, and to understand the pathophysiology of its deficiency.

## Author Summary

Glycosylation concerns the synthesis of sugar chains, their addition onto proteins and/or lipids, and their subsequent modifications. The resulting glycoproteins serve many critical roles in metabolism. The importance of this pathway is illustrated by a group of diseases called Congenital Disorders of Glycosylation (CDG). To date, over 60 distinct disorders have been described. In the present study, we demonstrated that mutations in *MAN1B1*, a gene formerly linked to non-syndromic intellectual disability, cause CDG. We described 7 patients with similar clinical features (developmental delay, intellectual disability, facial dysmorphism and obesity), defining MAN1B1-CDG as a syndrome. Furthermore, we confirmed that the MAN1B1 protein is localized into the Golgi apparatus instead of the endoplasmic reticulum, where it was assumed to reside for many years. Moreover, we showed that mutations in *MAN1B1* lead to alterations of the Golgi structure.

## Introduction

Congenital Disorders of Glycosylation (CDG) are a group of genetic diseases, due to deficient protein and lipid glycosylation [1]. To date, over 60 distinct disorders have been described, comprising a very broad range of phenotypes [2]. However, the search for the culprit gene in an unsolved CDG case can be very laborious, due to its heterogeneous clinical presentation and the extensive list of candidate genes. Over the last few years, massive parallel sequencing techniques have permitted to identify the underlying genetic defect in a growing number of diseases. In case of CDG, exome sequencing led to the discovery of 7 novel disorders in less than 2 years [3].

N-glycosylation of proteins is a meticulously orchestrated process occurring in the cytosol, the endoplasmic reticulum (ER) and the Golgi apparatus. Responsible for the modification of secreted and transmembrane proteins, N-glycosylation is considered as one of the most widespread forms of glycosylation. A prerequisite for export of a protein out of the ER and its further transport through the secretory pathway is the adaptation of a native folding state [4]. However, protein folding is inherently error prone. Therefore, a quality control system has evolved to ensure that only properly folded proteins reach the plasma membrane. Glycoproteins unable to acquire a correct conformation will be recognized as terminally misfolded and marked for degradation by demannosylation of their glycan moiety.

The α(1,2)-mannosidase, MAN1B1, catalyzes the removal of the terminal mannose residue from the middle branch of Man9GlcNAc2, hence generating a degradation signal corresponding to Man8GlcNAc2 isomer B [5]. Initially, MAN1B1 was predicted to function as an ER resident protein, based on the localization of its yeast orthologue Mns1p [6]. Also the overexpressed recombinant human orthologue was found to reside within the ER [7]. However, recent studies indicate that the endogenous MAN1B1 localizes to the Golgi apparatus of mammalian cells, implying that quality control is not confined to the ER, but stretches throughout the secretory pathway [8]. In this new model, MAN1B1 functions not only as a checkpoint for misfolded proteins escaping the ER, but also as a lectin retrieving these proteins back to the ER prior to their degradation by the 26S proteasome.

Interestingly, mutations in *MAN1B1* have been recently associated with non-syndromic autosomal recessive intellectual disability (NS-ARID) [9]–[10]. However, in the present study we show that MAN1B1 deficiency is associated with N-glycosylation disorders and causes a CDG-II syndrome. In addition, our findings suggest that MAN1B1 plays a role in protein quality control at the level of the Golgi apparatus.

## Results

### Whole exome sequencing and gene identification

To identify the causative gene in an unsolved CDG-II case, we performed whole exome sequencing by using the SeqCap EZ Human Exome Library v2.0 and the Illumina HiSeq2000. A total number of 29,518,319 reads were retrieved. 89% of the reads generated passed CASAVA quality filters, were unique, and mapped to the reference genome. The mean target coverage was 56, with 83% of the target covered at least 20-fold. Quality filtering led to a total of 29,437 variants, of those 11,026 were nonsynonymous. 702 variants had a frequency lower than 1% in the 1000 Genomes project. 8 homozygous variants and 132 compound heterozygous variants fitted with a recessive disease model. From this 140 variants, 4 were retained based on a Polyphen-2 score higher than 0.8. Out of this list, 2 were homozygous and 2 compound heterozygous (Table 1). The variant detected in *MAN1B1* (MIM 604346) encoded the c.1225T>C (p.S409P) mutation in a homozygous state. The sequencing depth of this variant was 22 reads. On the basis of the described function of *MAN1B1*, this gene was the best candidate from the list. Using Sanger sequencing, we confirmed the presence of the homozygous missense mutation in exon 8 of *MAN1B1*. This serine residue is highly conserved. The software tools SIFT, PolyPhen-2 and Project HOPE predicted the mutation to be damaging (Figure S1, Figure S2). The serine 409 is located within an alpha helix of the protein. We hypothesize that the mutation would impair MAN1B1 function by either breaking or kinking this helix.

**Table 1:** Overview of candidate variants identified by exome sequencing.

Gene symbol	Gene name	Transcript	Nucleotide change	Protein change	Mutation type	Predicted zygosity	Chromosome
*MAN1B1*	Mannosidase, alpha, class 1B, member 1	NM_016219	c.1225T>C	p.S409P	Missense	Homo	9
*WDR85*	WD repeat domain 85	NM_138778	c.824C>T	p.P275L	Missense	Homo	9
*CRIM1*	Cysteine rich transmembrane BMP regulator 1	NM_016441	c.618A>C	p.L206F	Missense	Het	2
*CRIM1*	Cysteine rich transmembrane BMP regulator 1	NM_016441	c.2104A>C	p.T702P	Missense	Het	2

% in the 1000 Genomes project were excluded. Based on recessive inheritance, a subsequent prioritization was applied using the following criteria: a minimum of 80% variant reads for potential homozygous and between 20 and 60% for compound heterozygous variants. For the analysis of candidate variants, variants from the single nucleotide polymorphism database (dbSNP) and with a higher frequency than 1

Sequencing of *MAN1B1* in a cohort of individuals with unsolved CDG-II allowed us to identify 6 other cases with *MAN1B1* mutations. Case 2 is compound heterozygous for the same missense mutation c.1225T>C (p.S409P) in exon 8, and the nonsense mutation c.172G>T (pE58X) in exon 1. Case 3 was found to carry the homozygous missense mutation c.1000C>T (p.R334C) in exon 7 (Figure S1). Cases 4.1 and 4.2 are homozygous for the same missense mutation as case 3, namely p.R334C. The arginine residue at position 334 is located within an alpha helix of the protein and strictly conserved (Figure S2). Furthermore it is localized at the active site pocket of the protein, where it interacts with the substrate via hydrogen bonding. The conversion to a cysteine might then alter the electrostatic interactions necessary for binding of the substrate in the active site. Case 5 was found to carry a homozygous 2 bp deletion c.1833_1834delAG (p.T611del), causing a frameshift that results in an alternative protein, missing many of the residues required for substrate recognition. Case 6 carried a heterozygous 4 bp deletion in intron 9 (c.1445+2delTGAG). Sequencing of full *MAN1B1* cDNA did not only confirm splicing of exon 9 due to the 4 bp deletion, but also revealed a heterozygous loss of exon 4. To prove that this event on cDNA was due to a deletion of exon 4, a long template PCR on genomic DNA stretching from exon 2 to exon 5 was performed. The PCR product revealed a shorter fragment of approximately 3,000 bp instead of the estimated 10,000 bp, confirming the presence of a heterozygous deletion of exon 4 (data not shown). Further sequencing of the boundaries showed a deletion of 6,453 bp (c.465+1460_620+527del). The 4 bp deletion in intron 9 was located on the maternal allele. The deletion of exon 4 occurred *de novo* on the paternal allele (Figure S1F).

### Clinical presentation

**Figure 1:**
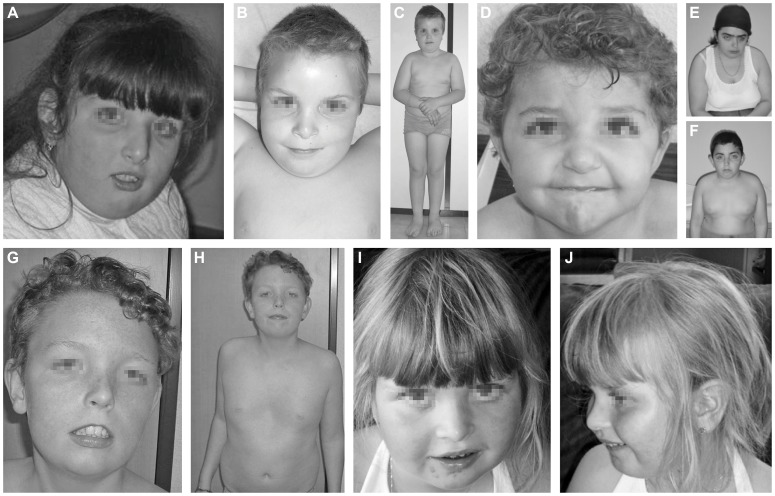
Clinical features of the seven cases. Clinical features of P1 at the age of 7 years (A), P2 at the age of 10 years (B, C), P3 at the age 3.5 years (D), P4.1 at the age of 18 years (E), P4.2 at the age of 12 years (F), P5 at the age of 13 years (G, H) and P6 at the age of 5 years (I, J). Note the facial dysmorphism, i.e. hypertelorism with downslanting palpebral fissures (A, D, E, F, G), large, low set ears (A, B, C, D, E, F, G, J), thin upper lip with hypoplastic nasolabial fold (A, B, D, E, F, G, I), tubular nose in P1, P4.1 and P4.2 (A, E, F) and a depressed nasal bridge in patients P2, P3, P5 and P6 (B, D, G, I). Note the truncular obesity (C, E, F, H) and the widely spaced, inverted nipples (B, F, H). Note the pectus excavatum in P5 (H).

The index case (P1, Figure 1A) is a 16 years old girl of Portuguese origin. Head circumference and length at birth were situated at percentile 97. The girl presented with psychomotor retardation and hypotonia. There was slight facial dysmorphism. A brain MRI performed at the age of 7, showed cerebellar hypoplasia with vermian atrophy. To date, she presents severe psychomotor retardation and obesity.

Case 2 (P2, Figure 1B, 1C) is a 12 years old boy. He was born as the first child of healthy, non-consanguineous parents of Portuguese origin. Developmental delay was noted at the age of 18 months. He presented with hypotonia and slight facial dysmorphism. Fingers were long and thin. Brain MRI was normal. To date, he displays moderate mental retardation (IQ 43) and obesity (BMI>>P97). Periods of auto-aggressive behaviour and repetitive movements occur.

Case 3 (P3, Figure 1D) is an 11 years old girl of Turkish origin. She presented with delayed motor development at the age of 3 years. Brain MRI was normal. She showed slight facial dysmorphism, hypermobility of the joints and skin laxity. To date she presents with mild mental retardation, short stature (length<P3), truncular obesity (weight at P50) and macrocephaly (head circumference at P97).

Case 4.1 (P4.1, Figure 1E) and case 4.2 (P4.2, Figure 1F) are siblings of Turkish origin. Parents are consanguineous (first cousins). Case 4.1 is a 24 years old woman. She came under medical attention because of mild psychomotor retardation and hypotonia. Brain MRI was normal. She showed slight facial dysmorphism, hypermobility of the joints and skin laxity. She presented an important scoliosis, which was corrected by surgery at the age of 18. To date, she presents mild mental retardation (IQ 62) and truncular obesity.

Case 4.2 is a 18 years old man. He presented with psychomotor retardation and hypotonia. Brain MRI was normal. There was only slight facial dysmorphism and skin laxity. To date, he presents mild mental retardation (IQ 69) and obesity.

Case 5 (P5, Figure 1G, 1H) is a 13 years old boy. He was born at term as the first child of healthy, non-consanguineous parents of Belgian origin. He presented with hypotonia and slight dysmorphic features. Fingers were long and thin. Hypermobility of the joints and skin laxity were noticed, as well as a pectus excavatum. Dilatation of the aortic root was seen on cardiac ultrasound. Brain MRI performed at the age of 3 months was normal. At the age of 5 years he was diagnosed with epilepsy (absences). To date, he presents mild mental retardation and is able to use simple language. Growth and weight evolution are normal, though he displays truncular fat accumulation.

Case 6 (P6, Figure 1I, 1J) is a 12 years old girl. She was born as the first child of healthy, non-consanguineous parents of Belgian origin. Reduced fetal movements were observed during pregnancy. After birth, she was diagnosed to have a muscular ventricular septum defect (VSD). Spontaneous closure occurred. Developmental delay was notified at the age of 18 months. She displayed hypotonia and a delay in motor development. There was slight facial dysmorphism, hypermobility of the joints, and skin laxity. Fingers were long and thin. At the age of 5, she was diagnosed with an autism spectrum disorder and mild mental retardation (IQ 52). Brain MRI showed multiple, small white matter lesions. Rapid bone maturation and early puberty occurred because of overweight.

Clinical features of all patients are furthermore compared in Table 2.

**Table 2:** Clinical features of patients with MAN1B1 deficiency.

Clinical features	P1	P2	P3	P4.1	P4.2	P5	P6
Current age (yrs)	16	12	11	24	18	13	12
Gender	F	M	F	F	M	M	F
Ethnicity	Portuguese	Portuguese	Turkish	Turkish	Turkish	Belgian	Belgian
Mental retardation	severe	moderate	mild	mild	mild	mild	mild
Delayed motor development	+	+	+	+	+	+	+
Behavioral problems	−	+	−	NA	NA	−	+
Autism	−	−	−	NA	NA	−	+
Seizures	−	−	−	−	−	+	−
Hypotonia	+	+	+	+	+	+	+
Cerebellar hypoplasia	+	−	−	−	−	−	−
Hypertelorism	+	−	+	+	+	+	+
Downslanting palpebral fissures	+	+/−	+	+	+	+	+
Large, low-set ears	+	+	+	+	+	+	+
Thin upper lip	+	+	+	+	+	+	+/−
Hypoplastic nasolabial fold	+	+	+	−	−	+	+
Truncal obesity	+	+	+	+	+	+	+
Inverted nipples	−	−	+	−	+	+	+
Joint hypermobility	−	−	+	+	−	+	+
Skin laxity	−	−	+	+	+	+	+

+) or absence (−) of each feature is reported for each individual. The following abbreviations are used: NA, not available; yrs: years; F: female; M: male. The presence (

**Figure 2:**
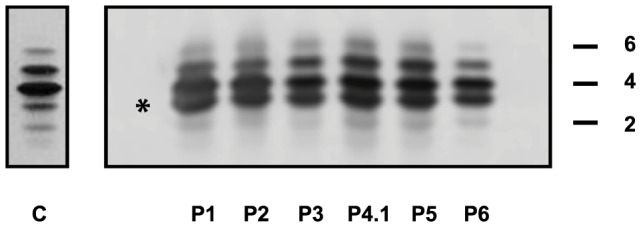
IEF profiles of serum transferrin. Isoelectrofocusing pattern of serum transferrin in MAN1B1-deficient individuals and a control. 2, 4 and 6 indicate disialo-, tetrasialo-, and hexasialotransferrin isoforms, respectively. The asterisk mark represents the trisialotransferrin isoform.

Capillary zone electrophoresis (CZE) of serum showed a type 2 transferrin pattern with an increase of trisialotransferrin (representing 31 to 41% of the transferrin isoforms, normal range: 1–8%) in all affected cases (Table S1). Isoelectrofocusing of serum transferrin (sTF-IEF) confirmed these results (Figure 2). This categorized them as CDG-II.

### Structural analysis of N-glycans and Golgi morphology

**Figure 3:**
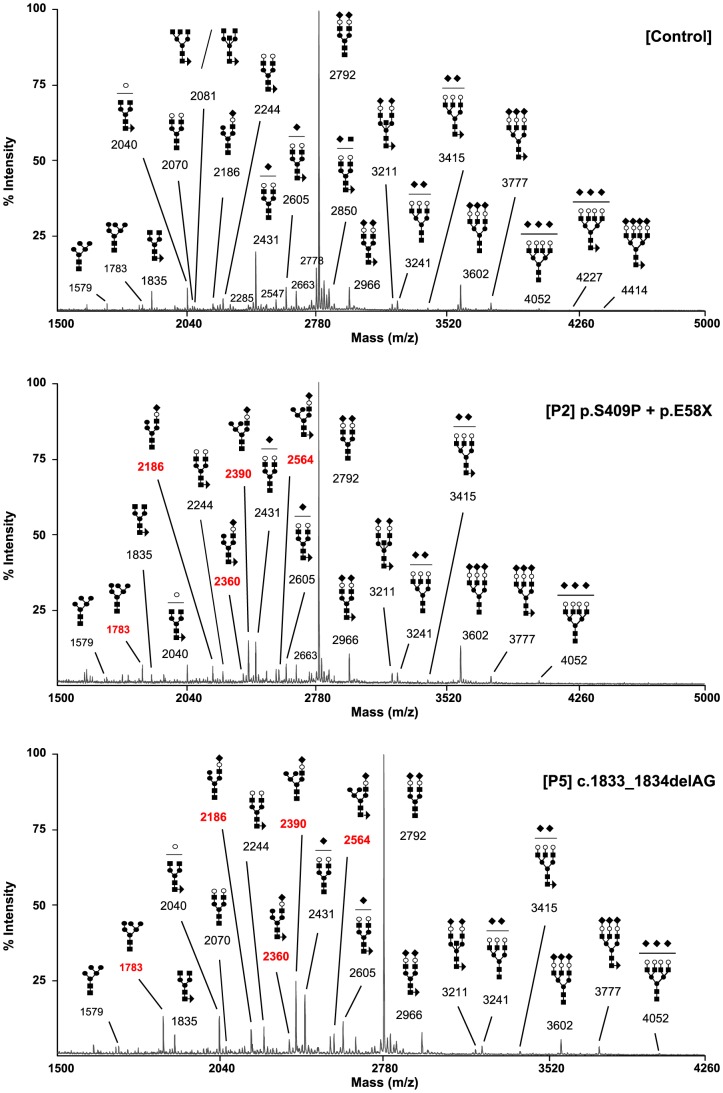
MAN1B1-deficient individuals are deficient in protein N-glycosylation. MALDI-TOF spectra of the permethylated N-glycans from sera of control and MAN1B1-deficient individuals. Respective m/z values of N-glycans structures accumulating in MAN1B1-deficient cells are displayed in red. The symbols representing sugar residues are as follows: closed square, N-acetylglucosamine; closed circle: mannose; open circle, galactose; closed diamond, sialic acid; and closed triangle, fucose. Linkages between sugar residues have been removed for simplicity.

The type 2 sTF-IEF pattern pointed to an N-glycan remodelling defect. Therefore, we investigated the Golgi glycosylation in more detail and determined the structures of the N-linked glycans on serum glycoproteins from controls and affected cases by using mass spectrometry (Figure 3). In MAN1B1-deficient individuals (P2, P5), we observed the accumulation of the hybrid-type glycan structure NeuAc1Hex6HexNAc3 (m/z 2,390), present together with the corresponding fucosylated NeuAc1Hex6HexNAc3dHex1 (m/z 2,564). We also observed a significant increase of the sialylated NeuAc1Hex5HexNAc3 (m/z 2,186) with respect to the control. This glycan also appeared to be present together with the corresponding fucosylated one (NeuAc1Hex5HexNAc3dHex1; m/z 2,360) which was not present in the control. In MAN1B1-CDG cases, there was also a marked increase of Hex6HexNAc2 (m/z 1,783), but no significant variations of Hex5HexNAc2 (m/z 1,579).

These results were confirmed in a second group of MAN1B1-CDG cases (P1, P4.1, P4.2 and P6) that were analysed independently (Figure S3). We noticed the presence of hybrid-type glycan structures NeuAc1Hex5HexNAc3 and NeuAc1Hex6HexNAc3. These glycans appeared to be present together with the corresponding fucosylated NeuAc1Hex5HexNAc3dHex1 and NeuAc1Hex6HexNAc3dHex1, respectively. These four species were not detected in controls.

**Figure 4:**
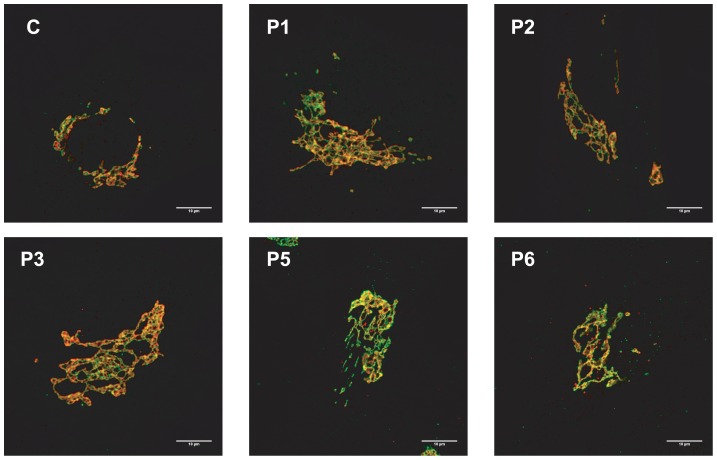
MAN1B1-deficient fibroblasts present alterations in Golgi structure. Golgi localization of GM130 (red) and TGN46 (green) in control and MAN1B1-deficient fibroblasts. Cells were fixed, double-labelled with antibodies against GM130 and TGN46, and analysed by confocal laser scanning microscopy. Scale bar represents 10 µm.

Because the N-glycans abnormalities suggested a Golgi glycosylation defect, Golgi morphology was investigated. Staining with the Golgi markers TGN46 and GM130 detected significant alterations in Golgi morphology in fibroblasts of all affected cases, with marked dilatation and fragmentation (Figure 4).

### Functional analysis of *MAN1B1* mutations

**Figure 5:**
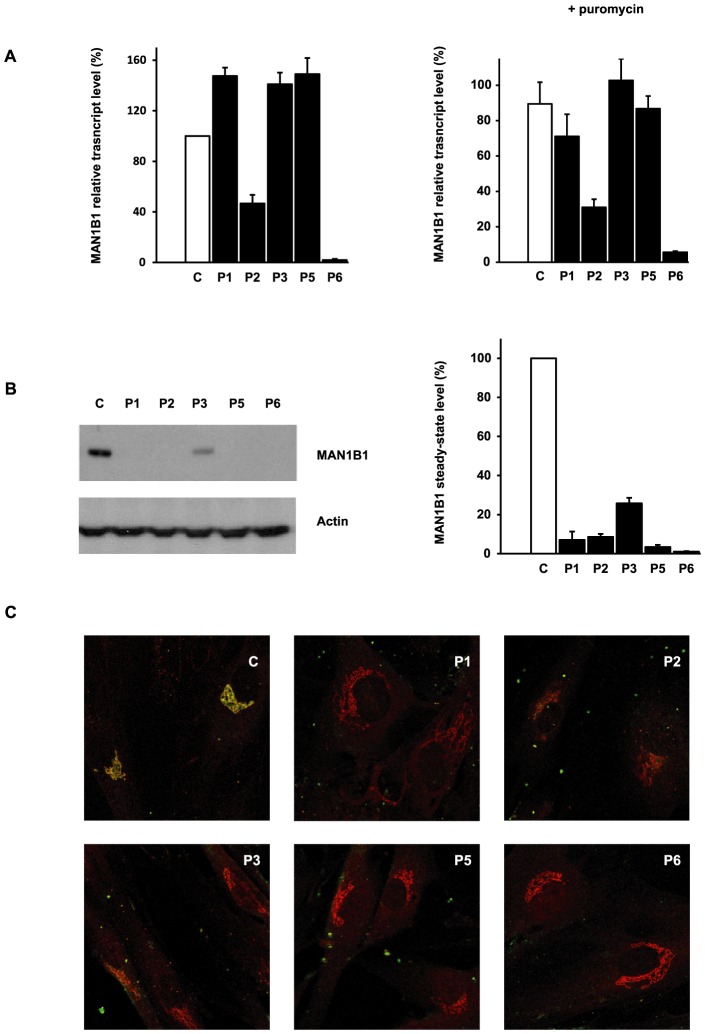
Functional analyses of *MAN1B1* mutations. (**A**) Quantification of the *MAN1B1* transcript in controls and affected individuals by qPCR without (left panel) and with (right panel) puromycin. *MAN1B1* expression was normalized to the expression of the house-keeping gene *HPRT*. Values plotted with a wild-type control untreated with puromycin are set to 1. The depicted values are the mean ± SEM of at least three independent experiments. (**B**) Steady-state levels of the expression of MAN1B1 in control (C) and MAN1B1-deficient (P) fibroblasts. Whole-cell extracts were analysed by immunoblotting using anti-MAN1B1 antibodies. Thirty micrograms of total cell extracts were loaded into each lane and anti-β-actin antibodies were used as a loading control. Quantifications represent the mean value of three independent loadings of three independent samples. (**C**) Intracellular distribution of MAN1B1 in control and MAN1B1-deficient fibroblasts. Fibroblasts were double-labelled with antibodies against MAN1B1 (green) and the Golgi marker giantin (red), then fixed and processed for analysis by confocal laser scanning microscopy. Images were collected under identical settings. Depicted are the zoomed images. The independent panels are presented in Figure S4.

We used quantitative real-time PCR (qPCR) to investigate the effects of the mutations on *MAN1B1* expression by comparing *MAN1B1* cDNA levels amplified from mRNA isolated from control and MAN1B1-deficient fibroblasts, cultured in the presence or absence of the translation inhibitor puromycin. Compared to controls, most of the MAN1B1-deficient individuals presented an expression increased 1.22 to 1.49 fold compared to the wild-type *MAN1B1* transcript. Case 2, who harbours the nonsense mutation p.E58X, showed an expected reduced expression level of 40% compared to control. Case 6 showed an expression level of 2% compared to control, likely because the deletions on both alleles lead to instability of the transcript (Figure 5A, left panel). Culturing control and MAN1B1-deficient fibroblasts in presence of puromycin did not markedly alter these expression levels (Figure 5A, right panel).

To further investigate the effects of the mutations on MAN1B1, we performed immunoblotting using control and MAN1B1-deficient fibroblasts. As shown in Figure 5B, an extremely low level of endogenous MAN1B1 was detected in affected cases. The steady-state level of MAN1B1 in P3 was reduced to 24%, suggesting that the protein was slightly more stable than in the other affected individuals.

We then compared the subcellular localization of MAN1B1 in control and MAN1B1-deficient fibroblasts by confocal microscopy. In accordance with the data recently published by Pan and coworkers [8], the endogenous MAN1B1 showed a clear perinuclear Golgi-like distribution (Figure 5C and Figure S4, first row) rather than a typical diffuse ER localization. As expected from the western blot analysis, some of the MAN1B1-deficient individuals did not show any staining. A faint Golgi localization could be seen in some of the affected cases, despite the largely predominant Golgi staining for giantin (Figure 5C, Figure S4).

### Subcellular MAN1B1 localization

**Figure 6:**
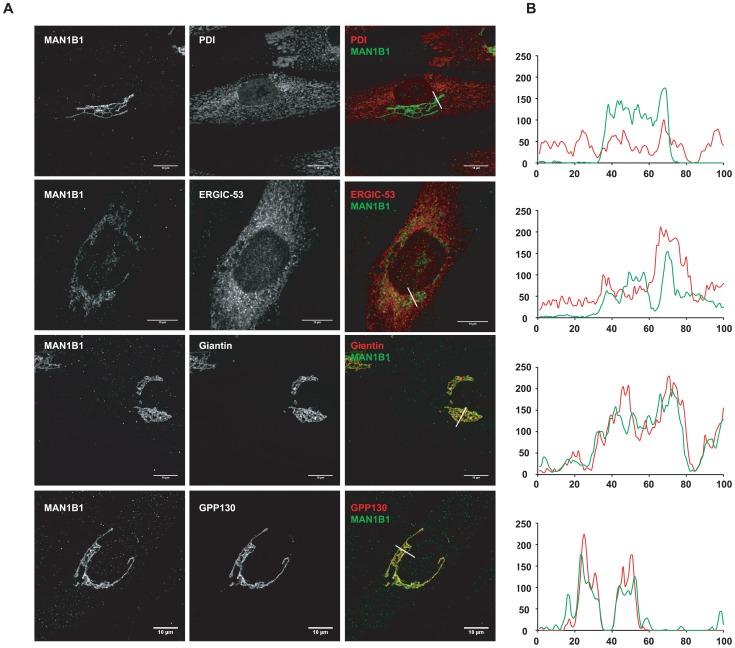
Subcellular localisation of MAN1B1. (**A**) Indirect double immunofluorescence staining of control and MAN1B1-deficient fibroblasts. The cells were double-labelled with antibodies against MAN1B1 and with antibodies against either the Golgi markers GPP130 and giantin, the ER-Golgi intermediate compartment (ERGIC) marker ERGIC-53, or the ER marker PDI. The cells were then examined by confocal laser scanning microscopy. Images were collected under identical settings. Scale bar represents 10 µm. (**B**) Confocal linescan analysis of the distribution of MAN1B1. The pixel intensity (vertical axis) of MAN1B1 (green) and PDI, ERGIC-53, giantin and GPP130 (from top to bottom, red) was hence measured along a vector drawn perpendicular across the Golgi stack and plotted versus distance (horizontal axis), using the RGB profiler plugin from Image J.

As mentioned above, MAN1B1 displayed a perinuclear Golgi distribution. Double labelling of control cells with MAN1B1 and the rough ER marker PDI showed a clear separation of the two proteins (Figure 6A). A similar pattern was obtained regarding the distribution of both MAN1B1 and the ER protein GRP78, suggesting that the mannosidase was located apart from the ER compartment (data not shown). In order to gain insights into the precise MAN1B1 subcellular localization, we examined its distribution in the Golgi and the ER-Golgi intermediate compartment (ERGIC). MAN1B1 showed a high level of colocalization with both the trans-Golgi marker GPP130 and the cis-Golgi marker giantin. Only weak colocalization with the ERGIC marker ERGIC-53 was noted (Figure 6A). The intensity of the different protein markers was plotted against the distance, using the RGB profiler plugin from Image J (Figure 6B). The level of colocalization was quantified as a percentage of the area occupied by the colocalized spots from the total labelled spots, based on Pearson's coefficient. These results showed a much higher degree of colocalization for MAN1B1 versus GPP130 (80%±3) or giantin (84%±2), than for MAN1B1 versus ERGIC-53 (41%±4), which is consistent with the qualitative data. These results strongly suggest that MAN1B1 is widely distributed within the Golgi apparatus and is hardly or not detectable in the ER.

### MAN1B1 deficient fibroblasts present a delay of trimming from Man9GlcNAc2 to Man8GlcNAc2

**Figure 7:**
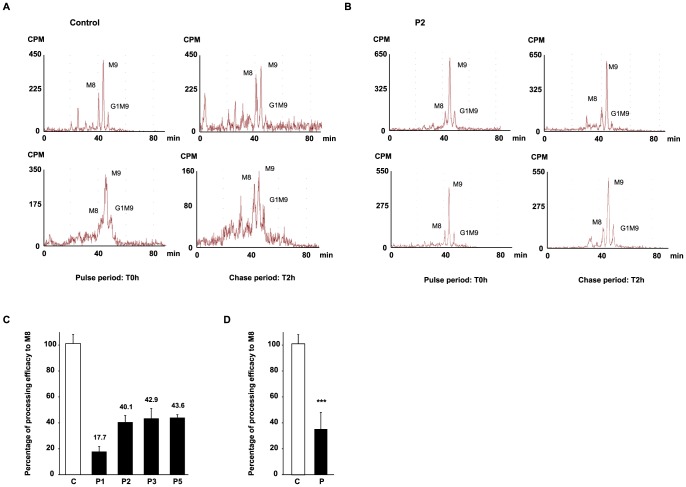
HPLC analysis of N-linked oligosaccharides from control and patient's fibroblasts reveal a delay of trimming from Man9GlcNAc2 to Man8GlcNAc2 in MAN1B1-deficient patients. Protein N-linked oligosaccharides of two different control (**A**) and one MAN1B1-deficient individuals (P2, **B**) were separated by HPLC after the pulse period (left panels) and 2 hours of chase (right panels). Depicted for both controls and P2 are two representative chromatograms from two independent experiments, with one set of experiments presented in the upper panel, and a second one in the lower panel. Symbols: M8, M9 indicate oligosaccharide species with 8 or 9 mannose residues and possessing two GlcNAc resides at their reducing ends. G1M9 indicates oligosaccharide species with 9 mannose and 1 Glc residues and possessing two GlcNAc residues at their reducing ends. (**C**) Relative peak areas of M8, M9, and G1M9 were quantified and the results expressed as the percentage of processing efficiency to M8. In the calculation, the trimming efficiency from M9 to M8, which corresponds to the difference between the percentage of M8 at T0 h and T2 h, was considered as 100%. The depicted values are the mean ± SEM of independent experiments. (**D**) Comparison of the mean processing efficacy of the three control cell lines (C) to the mean processing efficacy of all four MAN1B1-deficient cell lines (P). Data were analysed by using Student's t test. *** indicates a p value<0.001.

The impact of MAN1B1 on Golgi glycosylation was studied in MAN1B1-deficient fibroblasts. The cells of individuals mutated in MAN1B1 were further investigated by metabolic labelling. MAN1B1 is reportedly known to be the key enzyme responsible for the trimming of Man9GlcNac2 species to Man8GlcNAc2 species [5]. To address the potential deleterious effect of the mutations on MAN1B1 function, a structural analysis of the N-linked protein glycans was performed. Therefore, cells were pulse-labelled with 2-[^3^H] mannose and chased for 2 h. In healthy controls as well as in MAN1B1-deficient cells, Man8GlcNAc2, Man9GlcNAc2 and Glc1Man9GlcNAc2 structures were found after the pulse period (Figure 7A, 7B and Figure S5A). As expected, when the control glycoproteins were chased for 2 hours, an increase of Man8GlcNAc2 species was observed indicating that the Man9GlcNAc2 species can serve as substrate for further trimming by ER/Golgi mannosidases. In contrast, only a slight increase of Man8GlcNAc2 species was seen in MAN1B1-deficient cells (Figure S5B). The processing efficacy from Man9 to Man8 was hence estimated for both healthy controls and MAN1B1-deficient cells. Figure 7C shows that this processing efficacy was estimated to 100%±6.9 in control cells but ranged between 40.1%±5.9 to 43.6%±2.2 for MAN1B1-deficient cells. Surprisingly, this value was much lower for P1 cells, with an average efficacy of 17.7%±3.8. These results strongly suggest that in MAN1B1-deficient cells, a delay in the trimming of Man9GlcNAc2 species occurs. For statistical relevance, we compared the mean processing efficacy of the 3 control cell lines to the mean processing efficacy of all 4 MAN1B1-deficient cell lines (Figure 7D). We conclude that the deficiency of processing from Man9GlcNAc2 species to Man8GlcNAc2 species is not complete, since this value remains of 36.1%±12.8 (p value<0.001) in cells of affected individuals (Figure 7D). This is likely due to the action of other ER/Golgi mannosidases or alpha-mannosidase like proteins such as EDEM proteins, which enable trimming of Man9 to Man8 species in mammalian cells.

## Discussion

*MAN1B1* encodes the endoplasmic reticulum mannosyl-oligosaccharide 1,2-alpha-mannosidase (ERManI), which cleaves the terminal mannose from the middle branch of Man9GlcNAc2, then producing Man8GlcNAc2 isomer B. It is believed to play a key role in glycoprotein quality control, by targeting terminally misfolded proteins for ERAD. The capacity to distinguish between terminally misfolded proteins and properly folded intermediates as appropriate ERAD substrates is indeed known to be one of the early steps in protein quality control. Several studies in both yeast and mammalian cells already provided evidence that the removal of terminal mannose residues from asparagine-linked Man9GlcNAc2 glycan structures generates a signal for ERAD [11]. However, the exact function of MAN1B1 is still unclear.

In the present study, we definitely link mutations in *MAN1B1* to pathology that presents as a deficiency of glycosylation. The first patients with MAN1B1 deficiency were detected in a study on NS-ARID [9]. We identified *MAN1B1* mutations in 7 cases with unsolved CDG-II. All patients presented a certain degree of intellectual disability, but also had facial dysmorphism and obesity. Furthermore, some of them displayed joint hypermobility and skin laxity. Dysmorphic features were similar for all cases, i.e. downslanting palpebral fissures, hypertelorism, large low set ears, a hypoplastic nasolabial fold and a thin upper lip. These clinical characteristics correspond to the phenotype of the formerly published patients, although the author assigned the dysmorphism to family traits rather than to the disease [9]. Overall, individuals with MAN1B1-CDG present a mild phenotype, which is in concordance with studies in *Saccharomyces cerevisiae*, since the yeast orthologue *MNS1* is not essential for growth [12]. The mild disease resulting from MAN1B1 deficiency however contrasts with another defect of N-glycan trimming, which is caused by mutations in the glucosidase I (*MOGS*) gene. A single case of MOGS deficiency has been reported so far, characterized by dysmorphism and hypotonia, leading to the death of the affected infant [13]. We believe that MAN1B1 deficiency belongs to the relatively frequent CDG. In our cohort of solved CDG-II cases, the occurrence of MAN1B1-CDG is indeed over 25%, more frequent than TMEM165-CDG, COG5-CDG or COG7-CDG (14% respectively). From the overall number of solved CDG cases (CDG-I plus CDG-II) in our database, MAN1B1-CDG is the fifth most frequently encountered CDG type (1.9%), right behind SRD5A3-CDG (2.6%) or PMI-CDG (2.8%). PMM2-CDG and ALG6-CDG are the most frequent (69.9% and 8.5%, respectively). The genetic results were supported by a significant decrease in steady-state levels of the MAN1B1 protein. Enzymatic activity was measured indirectly by pulse-chase experiments with 2-[^3^H] mannose, which showed a significant delay in the trimming of Man9GlcNAc2 to Man8GlcNAc2. The trimming efficiency in MAN1B1-deficient cells was still up to 36%. This activity is likely due to the action of other ER/Golgi class I mannosidases or alpha- mannosidase-like proteins such as EDEM proteins, cleaving the terminal mannose residue at a lower rate.

Former studies localized the overexpressed recombinant MAN1B1 protein within the ER or the ERGIC. The *S. cerevisiae* homologue of MAN1B1, Mns1p, was originally demonstrated to function as an ER-resident protein [14]. The human *MAN1B1* was then identified and cloned on the basis of its close sequence homology with *MNS1* and was therefore predicted to localize and function in the ER [7] [15], despite a more than 50% sequence homology with Golgi α(1,2)-mannosidases IA, IB an IC [15]. We could clearly demonstrate that the endogenous MAN1B1 shows a Golgi distribution in primary skin fibroblasts. Further determination of its localization within the organelle revealed an equal distribution throughout the Golgi apparatus. This result confirms the initial observation of Sifers and coworkers, who used a panel of highly specific mAbs to demonstrate that human endogenous ERManI is O-glycosylated and localized in the Golgi apparatus [8]. As expected, only a faint or even no Golgi localization could be seen in MAN1B1-deficient cells.

One can wonder why the MAN1B1 protein, which is assumed to play a pivotal role in ERAD substrate generation, is physically separated from the ER. Traditionally, MAN1B1 was believed to function as a molecular clock in ER quality control. It was speculated that the slow processing activity of MAN1B1 determined the time needed for a protein to acquire its native folding state. In case of a terminally misfolded protein, futile folding cycles would provide MAN1B1 prolonged access to the glycan, resulting in its demannosylation and thereby marking the protein for proteasomal degradation [16]. However, recent findings indicate that the quality control system is not confined to the ER but stretches throughout the secretory pathway. In this model, MAN1B1 operates as a checkpoint within the Golgi apparatus, recycling misfolded proteins that escaped ERAD back to the ER, by interacting with the COP-I machinery. It is assumed that demannosylation of the glycan moiety will prevent the recycled protein from reentering the folding cycle after its retrieval to the ER. Instead, its degradation signal will be recognized by ER resident lectins, leading to proteasome mediated disposal of the misfolded protein [8].

How does MAN1B1 deficiency leads to disease? The purpose of quality control is to minimize the level and toxicity of misfolded proteins within the cell. So, failure of a checkpoint to recognize ERAD substrates might have serious repercussions on cell function. However, patients with MAN1B1-CDG present only a mild phenotype. Hence, one could assume that additional checkpoints exist in the Golgi apparatus. This hypothesis is supported by the observation that other Golgi α(1,2)-mannosidases were shown to be involved in glycoprotein quality control. In case of MAN1B1 deficiency, these Golgi mannosidases might be recruited for post-ER surveillance at the expense of their normal processing activity, leading to a delay in the trafficking of glycoproteins through the secretory pathway. If so, a relative accumulation of glycoproteins will occur within the Golgi apparatus prior to their impaired secretion. Storage of proteins will then activate a stress response increasing the capacity of the Golgi cisternae. The fact that MAN1B1 deficient cells display an altered Golgi morphology, characterized by dilatation and fragmentation of the cisternae, could support this hypothesis. However, this suggestion of a Golgi storage disease is in conflict with the model raised by Pan and coworkers, rather implying an increased secretion of misfolded glycoproteins in MAN1B1-depleted cells [8].

Furthermore, we believe that part of the phenotype might be explained by these Golgi abnormalities. First of all, disruption of Golgi morphology could explain the glycosylation deficiency observed in MAN1B1-deficient patients, by affecting the stability of key proteins responsible for the creation of the microenvironment necessary for proper Golgi glycosylation. Next, some of the observed clinical features (skin laxity, joint hypermobility) are also described in patients with COG5-, COG7- and ATP6VOA2-CDG. Interestingly, MAN1B1-deficient cells, as well as ATP6V0A2- and COG-deficient cells, show an altered Golgi morphology [17]–[18]. Furthermore, impaired secretion and intracellular retention of tropoelastin were already proven to result in skin laxity and joint hypermobility in patients with ATP6V0A2-CDG, implying that a similar mechanism could cause skin laxity in MAN1B1-deficient patients [19]. Finally, since the secretory pathway plays also an important role in neurotransmission and endocrine regulation, one can hypothesize that impaired protein secretion underlies the intellectual disability and obesity in our patients.

In conclusion, we have identified MAN1B1 deficiency as a relatively frequent CDG-II, since the occurrence of MAN1B1-CDG is over 25% in our cohort of unsolved CDG-II cases. Overall, the clinical picture is mild, comprising not only intellectual disability, but also truncal obesity and facial dysmorphism, hence defining MAN1B1-CDG as a syndrome. Furthermore, our results confirm that MAN1B1 is indeed localized to the Golgi apparatus. We hypothesize that part of the phenotype is linked to the disruption of Golgi morphology. Though, more work is needed to pinpoint the exact function of MAN1B1 in glycoprotein quality control and to understand the pathophysiology of its deficiency.

## Materials and Methods

### Ethics statement

Research on patients' cells was prospectively reviewed and approved by the Ethical Committee of the University Hospital of Leuven. PLOS consent forms were obtained for publication of clinical pictures.

### Capillary zone electrophoresis and isoelectric focusing of serum transferrin

Capillary zone electrophoresis (CZE) and isoelectric focusing of serum transferrin (sTF-IEF) were performed as previously described [20].

### Patients and cells

Primary fibroblasts from patients and controls were grown from a skin biopsy and cultured in Dulbecco's modified Eagle medium DMEM/F12 (Life Technologies) supplemented with 10% fetal bovine serum (Clone III, HyClones) at 37°C under 5% CO_2_.

### Exome sequencing

For the index case, mutation analysis was achieved by whole exome sequencing. Genomic DNA was sheared by sonication, platform-specific adaptors were ligated, and the resulting fragments were size selected. The library was captured using the SeqCap EZ Human Exome Library v2.0 (Roche NimbleGen), and 2× 76 bp paired-end reads were generated on a HiSeq2000 (Illumina). Reads that did not pass Illumina's standard filters were removed prior to alignment. Remaining reads were aligned to the reference human genome (hg19), using the CASAVA pipeline. Quality filtering was applied by excluding variants found in less than 5 reads and variants detected in less than 15% variant reads. Synonymous variants were excluded. To retrieve the quality-filtered private variants, those with a higher frequency than 1% in the 1000 Genomes project were excluded. Based on recessive inheritance, a subsequent prioritization was applied using the following criteria: a minimum of 80% variant reads for potential homozygous variants and between 20 and 60% for compound heterozygous variants. Variants with a Polyphen-2 score of less than 0.8 were filtered out.

### Sanger sequencing

Total RNA was isolated from the primary fibroblasts of the patient and control cells using the RNeasy Kit (Qiagen). The amount of extracted RNA was quantified using a NanoDrop spectrophotometer (Thermo Scientifics) and the purity of RNA was checked by the ratio of the absorbance at 260 and 280 nm. 2 µg of purified total RNA was then subjected to reverse transcription with the First-Strand cDNA synthesis Kit (GE Healthcare) following the manufacturer's instructions. For PCRs, cDNAs obtained after reverse transcription were diluted 1∶5.

Genomic DNA was extracted from white blood cells or fibroblasts from the patients and controls. Primers (available on demand) were designed to amplify the different exons of the *MAN1B1* gene (GenBank accession number NM_016219.4), including at least 50 bp of the flanking intronic regions. Consecutive primers within intron 3 and intron 4 were designed to find deletion breakpoints. Standard cDNA- and gDNA-based PCR reactions were based on 1 µl DNA in a total volume of 25 µl, 0.2 µl Platinum Taq polymerase (Invitrogen). Standard reaction conditions were 3 min at 95°C, then 10 cycles of 30 sec at 95°C, 30 sec at 65°C (−1°C each cycle), 2 min at 72°C, followed by 25 cycles of 30 sec at 95°C, 30 sec at 55°C and 2 min at 72°C. The reaction was finished by an incubation of 5 min at 72°C.

A long template PCR stretching from exon 2 to exon 5 was performed using the Expand Long Template PCR System (Roche Applied Science). The PCR reaction was based on 4 µl DNA in a total volume of 50 µl, 0.75 µl Expand Long Template Enzyme Mix, 5 µl of Buffer 2, a final 300 nM concentration of each primer and a final 500 µM concentration of dNTPs. Reaction conditions were 2 min at 95°C, then 10 cycles of 10 sec at 95°C, 30 sec at 65°C, 8 min at 68°C, followed by 25 cycles of 15 sec at 94°C, 30 sec at 65°C and 8 min (+20 sec each cycle) at 68°C. The reaction was completed with a final elongation of 7 min at 68°C.

For the sequencing of the resulting PCR product, the BigDye Terminator Ready reaction cycle sequencing kit v.3.1 (Applied Biosystems) was used. Analysis of the results was performed on an ABI3100 Avant (Applied Biosystems).

### Mass spectrometric analysis of N-linked glycans released from total plasma proteins

The glycans from total plasma N-glycoproteins were released as previously described [21]–[22]. Analysis of N-linked glycans on plasma glycoproteins was carried out by matrix-assisted laser desorption ionization time-of-flight mass spectrometry (Ciphergen) essentially as described for MALDI-TOF-MS, and performed in a positive linear ion mode [21]. The reference samples were anonymous plasma samples from patients without CDG and without a lysosomal storage disease. For cases P1, P4.1, P4.2 and P6, the lab of P. Mills and colleagues performed the analysis. For cases P2, P3 and P5, the lab of L. Sturiale and colleagues performed the analysis.

### Antibodies

Anti-MAN1B1 monoclonal antibodies used for indirect immunofluorescence were kindly provided by the lab of Richard N. Sifers and colleagues (Baylor College of Medicine, Houston, USA). Monoclonal anti-MAN1B1 antibodies used for western blotting were from Novus Biologicals. Polyclonal antibodies anti-GPP130 and anti-Giantin were purchased from Covance, polyclonal antibodies anti-PDI from Cell Signalling, polyclonal antibodies anti-ERGIC53 from Sigma-Aldrich and polyclonal anti-GRP78 BiP antibodies from Abcam.

### Immunoblotting

30 µg of proteins were analysed by SDS-PAGE and immunoblotted onto Hybond-ECL nitrocellulose membrane (Amersham Biosciences UK limited), with the indicated antibodies. Cells were rinsed twice with ice-cold phosphate buffer saline (PBS) and then lysed for 5 min on ice in cell lysis buffer (25 mM Tris-HCl, 150 mM NaCl pH 7.6, supplemented with 1% Triton X-100, 1% sodium deoxycholate and 0.1% SDS). Proteins were quantified by using the Micro BCA Protein BSA Assay Kit (Fischer Scientific).

Signals were detected using the Western Lightning-ECL, Enhanced Chemiluminescence Substrate (Perkin Elmer) according to the manufacturer's instructions. Signal detection was performed by autoradiography and quantified with the ImageQuant LAS 4000 (GE Healthcare), using the ImageQuant LAS 4000 Control Software for analysis.

### Immunofluorescence staining

Cells were grown on glass coverslips, washed once with PBS and fixed by incubation for 30 min with 4% paraformaldehyde in 0.1 M sodium phosphate buffer (pH 7.2) at room temperature. The coverslips were rinsed three times with PBS for 5 min. The fixed cells were then permeabilized with PBS containing 0.5% Triton X-100 for 5 min and washed three times with PBS. Next, the fixed cells were incubated for at least 60 min with a blocking solution containing 0.1% Triton X-100 (Sigma-Aldrich), 1% BSA (Roche Applied Science), and 5% normal goat serum (Life Technologies) in PBS. After blocking, the cells were incubated overnight at 4°C with primary antibodies diluted in the previously described blocking solution. The cells were then washed again three times, followed by incubation with the Alexa 488- or Alexa 568-conjugated secondary antibodies (1∶500, Life Technologies) for 1 h at room temperature in the dark.

Immunostaining was detected through an inverted Leica SP5 spectral microscope with a 63× oil immersion lens at room temperature. Data were therefore collected using the LAS 6000 AF software and finally processed in Adobe Photoshop 7.0 (Adobe systems). Colocalization was quantified using the colocalization plugin of ImageJ. The channel ratio was always set at 90%.

### Real-time quantitative PCR

PCRs were performed for the *MAN1B1* gene (GenBank NM_016219.4) and the house-keeping gene *HPRT* (Hypoxanthine PhosphoRibosylTransferase, GenBank NM_000194), which was used as an endogenous control for normalization. PCR primers were designed using Primer 3 software. All the primers were synthesized by Integrated DNA Technologies. PCR primers used are the followings: *MAN1B1* (5′-CTGTGGATCCCCGCCCGGAA-3′ and 5′-TCAGATGCACTGGTGTGCCCTGT-3′) and *HPRT* (5′-GCCAGACTTTGTTGGATTTG-3′ and 5′-CTCTCATCTTAGGCTTTGTAT TTTG-3′). PCRs (20 µl) were performed using 2× LightCycler 480 SYBR Green I Master (Roche Applied Science) with 2 µl of a 1∶5 cDNA dilution and 300 nM final concentration of each primer. Data were analysed using the LightCycler 480 Software (Roche Applied Science). The comparative threshold cycle method described by Livak and Schmittgen was used to quantify the results [23]. In addition, serial dilutions were used to create standard curves for relative quantification, and the expression of the *MAN1B1* transcript was normalized to *HPRT* expression.

### Metabolic labelling

Fibroblasts (8×10^6^ cells per labelling) were grown overnight in a 175 cm^2^ tissue culture flask. After 24 h, cells were preincubated for 45 min in 0.5 mM glucose and then pulse-radiolabelled for 2 h with 150 µCi of 2-[H^3^]-mannose (16 Ci.mmol^−1^, Amersham Biosciences) at the same glucose concentration. For chase experiments, the radioactive culture medium was replaced by a medium containing 5 mM glucose and 5 mM mannose. After metabolic labelling or chase period, cells were scraped with 1.1 ml MeOH/H_2_O (8∶3) followed by the addition of 1.2 ml CHCl_3_. Sequential extraction of oligosaccharide material was performed as previously described [24].

### Analysis of oligosaccharide material by HPLC

Glycoproteins fraction obtained at the end of the sequential extraction were digested overnight at room temperature with trypsin (1 mg.ml^−1^; Sigma-Aldrich) in 0.1 M ammonium bicarbonate buffer, pH 7.9. The resulting glycopeptides were treated with 0.5 U PNGase F (Roche Applied Science) in 50 mM phosphate buffer, pH 7.2 for 4 h to release the oligosaccharides from the peptides. They were subsequently desalted on Bio-Gel P2 columns and eluted with 5% acetic acid.

The oligosaccharides were afterwards separated by HPLC on an amino derivated Asahipak NH_2_P-50 column (250×4.6 mm; Asahi) applying a gradient of acetonitrile/H_2_O ranging from 70∶30 over 50∶50 over 90 min at a flow rate of 1 ml per min. Oligosaccharides were identified on the basis of their retention time, compared to standard glycans, as previously described [24]. Elution of the labeled oligosaccharides was monitored by continuous β-counting with a flo-one β detector (Packard).

### Calculation peak counts

HPLC chromatograms were analysed using the ProFSA software (Perkin Elmer). To compare the percentage of specific oligomannoside structures, a fixed amount (50,000 dpm) of radioactivity was injected into HPLC. The counts in each peak were calculated on the basis of the peak area and normalized against the total number of counts in the injected samples. Moreover, the differences in the number of mannose residues between the different oligomannoside structures were taken into account. Hence, the radioactivity associated to the Man5 species was multiplied by 9/5 to be comparable to the radioactivity associated to the Man9 species.

## Supporting Information

Figure S1 MAN1B1 mutations detected in the cohort. Chromatograms of patients P1, P2, P3, P4.1, P4.2 and P5 respectively (**A–F**). Left panel: maternal allele. Right panel: paternal allele. Upper sequence: wild type allele in a control. Bottom sequence: mutant allele in the patient. The base pair of interest is marked with *. Heterozygous variants are annotated as ‘N’. Concerning panel B: note the presence of the benign variant c.176A>G;p.N59S (#) both in the control as in patient P2. (**G**) Chromatograms of patient P6 on genomic DNA (upper panel) and cDNA (bottom panel). Left panel: maternal allele, showing a 4 bp deletion within intron 9, leading to skipping of exon 9. Right panel: de novo deletion of 6.453 bp on the paternal allele, displaying the intronic boundary. Exonic base pairs are displayed in capitals. Intronic base pairs are displayed in small cases. Please note, this file is 41.7MB.
(EPS)

Figure S2 Conservation of amino acid residue across species displayed for the described missense mutations p.S409P and p.R334C, respectively.
(EPS)

Figure S3 Predicted identity and relative amounts of glycans released from total serum N-glycoproteins. Abbreviations and symbols: n.d., not detected; closed square, N-acetylglucosamine; closed circle, mannose; open circle, galactose; closed diamond, sialic acid; and closed triangle, fucose.
(EPS)

Figure S4 Intracellular distribution of MAN1B1 in control and MAN1B1-deficient fibroblasts. Fibroblasts were double-labelled with antibodies against MAN1B1 (green) and the Golgi marker giantin (red), then fixed and processed for confocal laser scanning microscopy. Images were collected under identical settings. Scale bar represents 25 µm. Please note, this file is 49.8MB.
(EPS)

Figure S5 HPLC analysis of N-linked oligosaccharides from control and patient's fibroblasts. (**A**) Protein N-linked oligosaccharides of MAN1B1-deficient individuals (P1, P3, P5) were separated by HPLC after the pulse period (left panels) and 2 hours of chase (right panels). Symbols: M8, M9 indicate oligosaccharide species with 8 or 9 mannose residues and possessing two GlcNAc resides at their reducing ends. G1M9 indicates oligosaccharide species with 9 mannose and 1 Glc residues and possessing two GlcNAc residues at their reducing ends. (**B**) Relative peak areas of M8 (black), M9 (red) and G1M9 (blue) were quantified. Values are depicted as percentages of total peak areas. The depicted values are the mean ± SEM of independent experiments.
(EPS)

Table S1 CZE profile of serum transferrin. Distribution of the transferrin isoforms in both control and MAN1B1-deficient individuals. 3, 4, 5, and 6 respectively indicate trisialo-, tetrasialo-, pentasialo-, and hexasialotransferrin isoforms. Values are depicted as percentages of total transferrin.
(DOCX)
