# Supplementary material for: MAN1B1 Deficiency: An Unexpected CDG-II
Source: PLoS Genet. 2013 Dec 12;9(12):e1003989. doi: 10.1371/journal.pgen.1003989 (PMC3861123; doi:10.1371/journal.pgen.1003989)
Supplement: Table S1 — CZE profile of serum transferrin. Distribution of the transferrin isoforms in both control and MAN1B1-deficient individuals. 3, 4, 5, and 6 respectively indicate trisialo-, tetrasialo-, pentasialo-, and hexasialotransferrin isoforms. Values are depicted as percentages of total transferrin. (DOCX) [file pgen.1003989.s006.docx]

|  | **3** | **4** | **5** | **6** |
| --- | --- | --- | --- | --- |
| **C** | 2.1 | 72.8 | 10.8 | 1.9 |
| **P1** | 35.2 | 56.0 | 6.5 | 2.4 |
| **P2** | 33.4 | 54.9 | 9.9 | 1.9 |
| **P3** | 31.1 | 57.9 | 9.5 | 1.6 |
| **P4.1** | 39.2 | 51.6 | 8.2 | 1.1 |
| **P5** | 32.2 | 53.4 | 11.8 | 2.6 |
| **P6** | 41.2 | 55.1 | 3.7 | 0.0 |
